# Intravenous Tissue Plasminogen Activator in Combination With Mechanical Thrombectomy: Clot Migration, Intracranial Bleeding, and the Impact of “Drip and Ship” on Effectiveness and Outcomes

**DOI:** 10.3389/fneur.2020.585929

**Published:** 2020-12-09

**Authors:** Adam Chang, Elham Beheshtian, Edward J. Llinas, Oluwatoyin R. Idowu, Elisabeth B. Marsh

**Affiliations:** ^1^Department of Neurology, The Johns Hopkins School of Medicine, Baltimore, MD, United States; ^2^Department of Radiology, The Johns Hopkins School of Medicine, Baltimore, MD, United States

**Keywords:** MCA occlusion, thrombectomy, stroke, IV tPA, hemorrhage

## Abstract

**Purpose:** Intravenous tissue plasminogen activator (tPA) is indicated prior to mechanical thrombectomy (MT) to treat large vessel occlusion (LVO). However, administration takes time, and rates of clot migration complicating successful retrieval and hemorrhagic transformation may be higher. Given time-to-effectiveness, the benefit of tPA may vary significantly based on whether administration occurs at a thrombectomy-capable center or transferring hospital.

**Methods:** We prospectively evaluated 170 individuals with LVO involving the anterior circulation who underwent MT at our Comprehensive Stroke Center over a 3.5 year period. Two thirds (*n* = 114) of patients were admitted through our Emergency Department (ED). The other 33% were transferred from outside hospitals (OSH). Patients meeting criteria were bridged with IV tPA; the others were treated with MT alone. Clot migration, recanalization times, TICI scores, and hemorrhage rates were compared for those bridged vs. treated with MT alone, along with modified Rankin scores (mRS) at discharge and 90-day follow-up. Multivariable regression was used to determine the relationship between site of presentation and effect of tPA on outcomes.

**Results:** Patients presenting to an OSH had longer mean discovery to puncture/recanalization times, but were actually more likely to receive IV tPA prior to MT (70 vs. 42%). The rate of clot migration was low (11%) and similar between groups, though slightly higher for those receiving IV tPA. There was no difference in symptomatic ICH rate after tPA. TICI scores were also not significantly different; however, more patients achieved TICI 2b or higher reperfusion (83 vs. 67%, *p* = 0.027) after tPA, and TICI 0 reperfusion was seen almost exclusively in patients who were not treated with tPA. Those bridged at an OSH required fewer passes before successful recanalization (2.4 vs. 1.6, *p* = 0.037). Overall, mean mRS scores on discharge and at 90 days were significantly better for those receiving IV tPA (3.9 vs. 4.6, 3.4 vs. 4.4 respectively, *p* ~ 0.01) and differences persisted when comparing only patients recanalized in under 6 h.

**Conclusion:** Independent of site of presentation, IV tPA before MT appears to lead to better radiographic outcomes, without increased rates of clot migration or higher intracranial hemorrhage risk, and overall better functional outcomes.

## Introduction

In 1995, intravenous tissue plasminogen activator (tPA), a thrombolytic designed to recanalize occluded blood vessels, was shown to significantly improve outcomes and became the first FDA approved treatment for acute ischemic stroke (1). Subsequent studies have shown that following IV tPA with mechanical thrombectomy (MT) in patients with large vessel occlusion (LVO) [e.g., internal carotid (ICA) terminus or middle cerebral artery (MCA) lesions] can improve outcomes further (2–5). This has become the standard practice at most Comprehensive Stroke Centers.

In trials demonstrating the efficacy of MT for large vessel occlusion, the majority of eligible patients were bridged with IV tPA prior to intervention (2–5). Interestingly, in 2013, the SYNTHESIS Expansion Investigators compared treatment with IV tPA plus MT to MT alone and found them to be equivalent (6). It has been proposed that tPA, which works immediately on clot breakdown, may facilitate the use of MT and help to treat distal embolization (7, 8); however, a recent study published in *Stroke* suggested that the use of IV tPA prior to mechanical lysis may complicate the procedure by converting easy to reach clots located at the proximal M1 branch to distal M2 branches (9). This, phenomenon could account for the unanticipated results of the SYNTHESIS trial and were not evaluated as part of the study. Additionally, though not different in the SYNTHESIS trial, it is possible that in some cases IV tPA may increase the rate of other complications such as hemorrhagic transformation that worsen long-term outcomes.

In this study, we evaluate the positive and negative effects of IV tPA administration prior to MT for patients presenting with LVO of the anterior circulation, and their potential impact on long-term outcomes. Individuals who received IV tPA are compared to those who did not due to either a medical contraindication (e.g., on systemic anticoagulation) or because they presented outside the 4.5 h treatment window. We stratify individuals by site of presentation [our local Emergency Department (ED) vs. transfer from an outside hospital (OSH)] to account for the effect of time on tPA's efficacy, and determine the rate of distal clot migration after combined treatment (IV tPA and MT) compared to MT alone. We then evaluate the effect of IV tPA on the efficiency and success of mechanical thrombectomy (time to recanalization and degree of reperfusion [Thrombolysis in Cerebral Infarction (TICI) score)] and risk of hemorrhagic transformation. A subgroup analysis is performed comparing functional outcomes for only those recanalized in under 6 h, to account for potential bias due to treatment in later time windows. Results will further inform the risk-benefit discussion when considering treatment options for acute ischemic stroke.

## Materials and Methods

This study was approved by the Johns Hopkins Institutional Review Board, who waived the need for informed consent given the observational nature of the study. A cohort of patients presenting directly to the Emergency Department at our large, urban, Comprehensive Stroke Center, or transferred from an outside hospital, between January 2016 and June 2019 who underwent MT for acute stroke due to LVO was prospectively followed. Mechanical thrombolysis was performed using either mechanical aspiration (MAT) or a stentriever device (SMAT). Intra-arterial tPA was rarely administered during intervention. Similar to prior MT trials, only individuals with thrombus involving the internal carotid artery (ICA) terminus, middle cerebral artery (MCA), and anterior cerebral artery (ACA) were included in the final analysis. Eligible patients presenting within 4.5 h of symptom onset (10) were treated with IV tPA prior to MT based on current practice guidelines (11).

Patient demographics (age, race, sex), medical variables (baseline modified Rankin score (mRS) (12), history of atrial fibrillation, diabetes mellitus, smoking, home medications), stroke characteristics [time of stroke discovery, stroke etiology (by TOAST criteria) (13), infarct volume, admission NIH Stroke Scale (NIHSS) score (14)], intervention-related variables [time of groin puncture and recanalization, ASPECTS score (15), collateral grade (16), thrombolysis modality (aspiration vs. stentriever), number of passes to achieve reperfusion of TICI 2b or greater, final TICI score (17)], and outcomes (length of stay, mRS at discharge and 90 day follow-up, 90 day mortality) were recorded.

### Clot Migration

Thrombus location was confirmed by a board certified neuroradiologist using the patient's initial MRA or CTA and compared to clot location on digital subtraction angiography at the time of thrombectomy. Clot migration was defined as movement of the thrombus: (1) to a more distal named vessel (e.g., ICA to MCA), (2) from the proximal to distal M1 branch of the MCA, or (3) to a distal branch of a major vessel (e.g., M1 to M2 segments).

### Hemorrhagic Transformation and Infarct Volume

The clinical course was reviewed by a board-certified vascular neurologist. Evidence of blood on follow-up imaging (non-contrast head CT or MRI) within 36 h of initial treatment, along with a change in examination of 4 NIHSS points or more was considered a symptomatic intracranial hemorrhage (18). Final infarct volume was calculated from the patient's MRI using the Generic Lesion Segmentation tool in Carestream Vue PACS, version 12 (Carestream Vue PACS, 2019).

### Statistical Analysis

Analyses were performed using STATA version 14. Differences between patients presenting directly to our ED vs. an OSH were determined using Student's *t*-tests and chi square analysis for continuous and categorical variables, respectively. The groups were then analyzed separately and divided into those treated and not treated with IV tPA. Primary variables of interest included: rate of clot migration, hemorrhagic transformation, and mRS on discharge and at 90 days post-stroke. Functional outcomes were also reported as “good” (mRS 0-2) vs. “poor” (mRS 3-6). Groin puncture to recanalization time, number of passes, and final TICI scores were also compared. Following univariate analysis, the effect of bridging with IV tPA on long-term outcome was adjusted for age, race, sex, baseline mRS, site of presentation (ED vs. OSH), collateral grade, and time from symptom onset to recanalization in multivariable linear regression. Regression analyses were also performed to look at independent predictors of clot migration, sICH, and 90-day mortality. To account for potential time-to-treatment bias, a subsequent sub-group analysis was performed comparing discharge and 90 day outcomes for only those patients recanalized within the early (<6 h) time window.

## Results

One hundred ninety patients were admitted and underwent MT at our Comprehensive Stroke Center over the 3.5 year study period; 170 had occlusions involving the anterior circulation and were included in further analysis. Approximately half (*n* = 87) were eligible and bridged with IV tPA prior to MT while the other 49% presented outside of the 4.5 h treatment window (*n* = 44, 53%) or did not meet inclusion criteria (on anticoagulation: *n* = 27, 33%; other: *n* = 12, 14%) (1). The majority of patients had high ASPECTs scores, but relatively poor collaterals, and were treated with stentrievers. Characteristics of the entire cohort are displayed in Table 1. The average age of the entire cohort was 69.5 years (SD 16.7). Forty-four percent were male; 32% were black. The mean infarct volume was 62.7 cc (SD 71.9). The average NIHSS on admission was 15.7 (SD 6.9) and the majority of strokes were due either to large artery disease (25%) or cardioembolism (62%) (13). The mean mRS at discharge was 4.3 (SD 1.6), and at 90 days was 3.9 (SD 2.1).

**Table 1:** Patient characteristics.

	**Total population**	**Emergency department**	**Outside hospital**	***P*-value**
	**(*N* = 170)**	**(*N* = 114)**	**(*N* = 56)**	
**Demographics**				
Age, mean years (SD)	69.5 (16.7)	69.7 (15.9)	69.0 (18.4)	0.813
Sex, *n* male (%)	74 (44)	50 (44)	24 (43)	0.901
Race, *n* black (%)	52 (32)	36 (32)	16 (30)	0.773
Ethnicity, *n* Hispanic (%)	6 (4)	4 (4)	2 (4)	0.894
**Medical characteristics**				
Baseline mRS, mean (SD)	0.6 (0.8)	0.5 (0.8)	0.6 (0.7)	0.510
Diabetes, *n* (%)	55 (32)	34 (30)	21 (38)	0.315
Hyperlipidemia, *n* (%)	81 (48)	58 (51)	23 (41)	0.229
Hypertension, *n* (%)	141 (83)	96 (84)	45 (80)	0.530
Atrial fibrillation, *n* (%)	85 (50)	55 (49)	30 (54)	0.549
Tobacco use, *n* (%)	74 (45)	51 (45)	23 (43)	0.834
Antiplatelet use, *n* (%)	54 (40)	35 (39)	19 (41)	0.824
Anticoagulant use, *n* (%)	31 (23)	19 (22)	12 (24)	0.724
**Stroke characteristics**				
IV tPA, *n* (%)	87 (51)	48 (42)	39 (70)	**0.001**
NIHSS on presentation, mean (SD)	15.7 (6.9)	15.2 (7.0)	16.7 (6.6)	0.185
ASPECTS, mean (SD)	9.1 (1.2)	9.3 (1.0)	8.6 (1.5)	**<0.001**
Collateral grade, *n* (%)				0.168
0	57 (34)	35 (31)	22 (39)	
1	105 (62)	75 (66)	30 (54)	
2	4 (2)	1 (1)	3 (5)	
3	4 (2)	3 (3)	1 (2)	
Stroke volume, mean (SD)	62.7 (71.9)	55.8 (57.4)	78.2 (95.8)	0.082
Stroke etiology, *n* (%)				0.688
Large artery	42 (25)	28 (25)	14 (25)	
Cardioembolism	104 (62)	68 (60)	36 (65)	
Small vessel	2 (1)	1 (1)	1 (2)	
Other etiology	8 (5)	7 (6)	1 (2)	
Undetermined	12 (7)	9 (8)	3 (5)	
**Intervention characteristics**				
Clot location (scan), *n* (%)				0.271
ICA	51 (30)	33 (29)	18 (33)	
M1	88 (52)	57 (50)	31 (56)	
M2	30 (18)	24 (21)	6 (11)	
Location (angio), *n* (%)				0.765
None	3 (2)	3 (3)	0 (0)	
ICA	48 (28)	30 (26)	18 (32)	
M1	78 (46)	54 (47)	24 (43)	
M2	40 (24)	26 (23)	14 (25)	
Clot migration, *n* (%)	18 (11)	10 (9)	8 (15)	0.254
Discovery to puncture, mean min (SD)	258.1 (196.5)	232.8 (187.7)	314.2 (206.0)	**0.021**
Discovery to recanalization, mean min (SD)	322.8 (244.7)	306.7 (251.5)	359.3 (227.6)	0.278
Door to scan, mean min (SD)		74.8 (60.4)		
Door to puncture, mean min (SD)		170.3 (140.2)		
Door to recanalization, mean min (SD)		229.6 (156.0)		
Puncture to recanalization, mean min (SD)	53.0 (33.5)	55.6 (34.0)	47.7 (32.1)	0.201
Modality, *n* (%)				0.644
MAT	16 (9)	12 (11)	4 (7)	
SMAT	130 (76)	88 (77)	42 (75)	
No. passes, mean (SD)	2.0 (1.4)	2.1 (1.5)	1.9 (1.2)	0.429
TICI score, *n* (%)				0.544
0	9 (6)	7 (7)	2 (4)	
1	3 (2)	3 (3)	0 (0)	
2a	12 (8)	10 (9)	2 (4)	
2b	46 (30)	30 (28)	16 (36)	
3	82 (54)	57 (53)	25 (56)	
Symptomatic ICH, *n* (%)	23 (14)	14 (13)	9 (16)	0.540
**Outcomes**				
Length of stay,mean days (SD)	9.5 (8.3)	10.3 (8.8)	7.9 (6.9)	0.079
mRS at discharge, mean (SD)−120 people	4.3 (1.6)	4.3 (1.6)	4.4 (1.7)	0.800
mRS at 90 days, mean (SD)−124 people	3.9 (2.1)	3.8 (2.0)	4.3 (2.3)	0.172
Mortality	48 (28)	31 (27)	17 (30)	0.667

*Bold values indicates statistical significance p < 0.05*.

### Effect of Site of Presentation

Patients presenting to the ED were similar at baseline to those transferred from an OSH with the exception of longer times from discovery to scan, groin puncture, and recanalization (see Table 1). Despite this, they were more likely to be treated with IV tPA prior to MT (70 vs. 42%, *p* = 0.001). Outcomes following intervention were also similar between groups.

### Effect of IV tPA

Though similar with respect to demographics and medical comorbidities, patients treated with IV tPA prior to thrombectomy were less likely to be on an anticoagulant prior to admission (7 vs. 38%, *p* < 0.001; see Table 2).

**Table 2:** Effect of IV tPA.

	**Emergency department**			**Outside hospital transfer**		
	**No tPA (N = 66)**	**tPA (*N* = 48)**	***P*-value**	**No tPA (*N* = 17)**	**tPA (*N* = 39)**	***P*-value**
**Demographics**						
Age, mean years (SD)	71.1 (15.6)	67.6 (16.2)	0.249	68.5 (16.5)	69.2 (19.3)	0.897
Sex, *n* male (%)	28 (42)	22 (46)	0.717	6 (35)	18 (46)	0.450
Race, *n* black (%)	20 (31)	16 (35)	0.656	4 (24)	12 (33)	0.468
Ethnicity, *n* Hispanic (%)	2 (3)	2 (4)	0.740	0 (0)	2 (6)	0.300
**Medical characteristics**						
Baseline mRS, mean (SD)	0.7 (0.8)	0.4 (0.6)	0.078	0.5 (0.7)	0.7 (0.7)	0.521
Diabetes, *n* (%)	26 (39)	8 (17)	**0.009**	2 (12)	19 (49)	**0.009**
Hyperlipidemia, *n* (%)	38 (58)	20 (42)	0.093	7 (41)	16 (41)	0.992
Hypertension, *n* (%)	61 (92)	35 (73)	**0.005**	15 (88)	30 (77)	0.327
Atrial fibrillation, *n* (%)	36 (55)	19 (40)	0.097	10 (59)	20 (51)	0.603
Tobacco use, *n* (%)	31 (48)	20 (42)	0.525	10 (59)	13 (36)	0.119
Antiplatelet use, *n* (%)	24 (45)	11 (31)	0.163	7 (50)	12 (38)	0.428
Anticoagulant use, *n* (%)	18 (34)	1 (3)	**0.001**	8 (50)	4 (12)	**0.004**
**Stroke characteristics**						
NIHSS on admission, mean (SD)	16.1 (6.7)	13.9 (7.3)	0.093	17.6 (7.4)	16.3 (6.3)	0.493
ASPECTS, mean (SD)	9.1 (1.1)	9.6 (0.7)	**0.004**	8.5 (1.4)	8.7 (1.5)	0.609
Collateral grade, *n* (%)			0.376			0.492
0	23 (35)	12 (25)		6 (35)	16 (41)	
1	42 (64)	33 (69)		11 (65)	19 (49)	
2	0 (0)	1 (2)		0 (0)	3 (8)	
3	1 (2)	2 (4)		0 (0)	1 (3)	
Stroke volume, mean (SD)	58.2 (61.8)	52.7 (51.7)	0.636	79.1 (84.7)	77.7 (102.3)	0.965
Stroke etiology, *n* (%)			0.37			0.604
Large artery	16 (25)	12 (25)		4 (24)	10 (26)	
Cardioembolism	41 (63)	27 (56)		13 (76)	23 (61)	
Small vessel	0 (0)	1 (2)		0 (0)	1 (3)	
Other etiology	5 (8)	2 (4)		0 (0)	1 (3)	
Undetermined	3 (5)	6 (13)		0 (0)	3 (8)	
**Intervention characteristics**						
Clot location (scan), *n* (%)			0.721			0.943
ICA	21 (32)	12 (25)		6 (35)	12 (32)	
M1	32 (48)	25 (52)		9 (53)	22 (58)	
M2	13 (20)	11 (23)		2 (12)	4 (11)	
Location (angio), *n* (%)			0.739			0.945
None	0 (0)	3 (6)				
ICA	19 (29)	11 (23)		6 (35)	12 (31)	
M1	30 (45)	24 (50)		7 (41)	17 (44)	
M2	16 (24)	10 (21)		4 (24)	10 (26)	
Clot migration, *n* (%)	5 (8)	5 (10)	0.597	2 (12)	6 (16)	0.696
Discovery to puncture, mean min (SD)	234.9 (154.6)	229.8 229 (2)	0.895	431.8 (322.5)	255.4 (58.7)	**0.005**
Discovery to recanalization, mean min (SD)	291.1 (157.1)	326.4 (337.0)	0.527	483.6 (365.9)	299.6 (70.2)	**0.019**
Door to scan, mean min (SD)	75.9 (67.3)	73.3 (50.1)	0.828			
Door to puncture, mean min (SD)	181.2 (153.7)	155.6 (119.6)	0.366			
Door to recanalization, mean min (SD)	242.4 (165.4)	213.3 (143.4)	0.386			
Puncture to recanalization, mean min (SD)	53.0 (31.1)	58.9 (37.5)	0.418	53.8 (39.1)	45.0 (28.9)	0.417
Modality, *n* (%)			0.291			0.771
MAT	6 (9)	6 (13)		1 (6)	3 (8)	
SMAT	52 (79)	36 (75)		13 (76)	29 (74)	
No. passes, mean (SD)	2.2 (1.5)	2.0 (1.4)	0.524	2.4 (1.5)	1.6 (0.9)	**0.037**
TICI score, *n* (%)			0.197			0.189
0	6 (10)	1 (2)		2 (13)	0 (0)	
1	2 (3)	1 (2)		0 (0)	0 (0)	
2a	8 (13)	2 (4)		1 (7)	1 (3)	
2b	15 (25)	15 (32)		4 (27)	12 (40)	
3	29 (48)	28 (60)		8 (53)	17 (57)	
Sympomatic ICH, *n* (%)	9 (14)	5 (10)	0.543	4 (24)	5 (13)	0.316
**Outcomes**						
Length of stay, mean days (SD)	11.1 (9.9)	9.3 (7.1)	0.282	9.2 (9.7)	7.3 (5.4)	0.372
mRS at discharge, mean (SD)−120 people	4.7 (1.4)	3.7 (1.7)	**0.004**	4.4 (1.6)	4.3 (1.8)	0.829
mRS at 90 days, mean (SD)−124 people	4.2 (1.9)	3.1 (2.1)	**0.009**	4.8 (1.9)	4.0 (2.5)	0.307
Mortality, *n* (%)	23 (35)	8 (17)	**0.031**	7 (41)	10 (26)	0.245

*Bold values indicates statistical significance p < 0.05*.

### Clot Migration, Ease of Intervention, and Hemorrhagic Transformation

The overall rate of clot migration was low (11%). Distal migration did occur more frequently in patients after IV tPA (13 vs. 8%), though the difference did not reach statistical significance. For patients presenting directly to our ED, tPA administration did not increase door to groin puncture or recanalization times. These times were not calculated for those being transferred from an OSH given that they were taken immediately to the Interventional Radiology Suite for the procedure and had already received tPA. Data regarding the effect of tPA administration on transfer times were unavailable. There was also no significant difference in groin puncture to recanalization time for ED or OSH patients regardless of bridging. However, the percentage of patients achieving a TICI score of 2b or better was substantially higher for those bridged with IV tPA (83 vs. 67%, *p* = 0.027), and TICI 0 perfusion was seen almost exclusively in patients who were not treated with IV tPA. None of our selected variables were predictive of clot migration in our cohort, likely because the rate was low. Rate of symptomatic hemorrhage was not statistically different between groups, but tended to be higher for transferred patients who did not receive IV tPA. Patients were significantly more likely to experience a sICH if they were white (18 vs. 6%, *p* = 0.038) and had larger infarct volumes (58 vs. 103 cc, *p* = 0.018). These variables approached statistical significance in multivariable models adjusting for age, time from symptom discovery, and admission NIHSS. Variables including advanced age, hypertension, atrial fibrillation, higher TICI scores, the use of stentrievers as opposed to aspiration, and longer times to recanalization led to higher rates of sICH, but did not meet statistical significance even in univariate analysis. Not surprisingly, 90-day mortality was significantly higher for those with sICH. Similar factors were associated with increased risk for mortality as with sICH; however, in multivariable regression, only baseline mRS and infarct volume remained significant.

### Functional Outcomes

**Figure 1:**
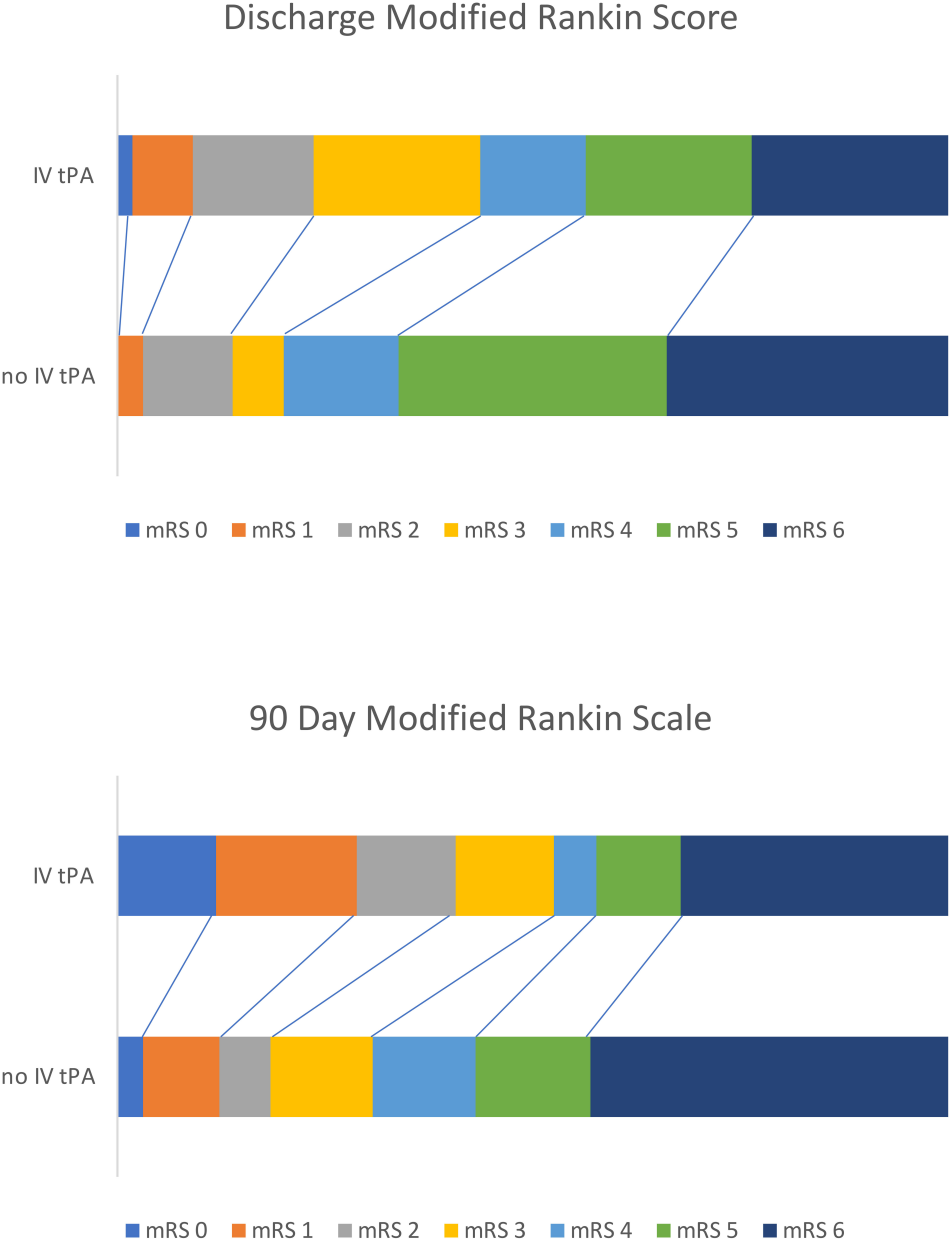
Rankin shift illustrating improved outcomes for those bridged with IV tPA before mechanical thrombectomy at both discharge and 90 days post-stroke.

Patients treated with IV tPA plus MT experienced a better overall functional recovery, with significantly lower mRS scores at both discharge [3.9 (SD 1.7) vs. 4.6 (SD 1.4), *p* = 0.011] and follow-up [3.4 (SD 2.3) vs. 4.4 (SD 1.9), *p* = 0.012] compared to those only receiving MT. A Rankin Shift is displayed in Figure 1 illustrating improved outcomes at both discharge and 90 days post-stroke for patients bridged with IV tPA prior to mechanical thrombectomy. When comparing good (mRS 0-2) vs. poor (mRS 3-6) functional outcomes, those treated with IV tPA were more likely to have a good outcome at both discharge (24 vs. 14%, *p* = 0.167) and 90 days (41 vs. 18%, *p* = 0.006), but only long-term results reached significance. While functional outcomes differed, final infarct volumes and length of stay were similar between the two groups. Results did not change when evaluating only patients recanalized within the early (<6 h) treatment window (*n* = 73). Patients treated with IV tPA prior to MT within this window continued to demonstrate lower mRS scores at discharge [3.9 (SD 1.8) vs. 4.9 (SD 1.3), *p* = 0.010] and 90 days [3.3 (SD 2.4 vs. 4.7 (SD 1.6), *p* = 0.007] compared to those treated with MT alone.

Results from the multivariable linear regression are displayed in Table 3. The group bridged with IV tPA was found to have better mRS scores at discharge and 90-day follow-up than those treated with MT alone even when age, race, sex, site of presentation, collateral grade, baseline mRS, and time to recanalization, were adjusted for. Age, collateral grade, and baseline mRS were also found to be independently associated with improved outcome. Results were most significant at 90-day follow-up.

**Table 3:** Multivariable regression of discharge and 90 day post-stroke outcomes.

	**Discharge mRS**			**90 day mRS**		
	**Coefficient**	***P*-value**	**95% Confidence interval**	**Coefficient**	***P*-value**	**95% Confidence interval**
IV tPA	−0.514	0.069	−1.068 to 0.040	−0.775	**0.045**	−1.531 to −0.019
Age	0.036	**<0.001**	0.018–0.055	0.049	**<0.001**	0.024–0.073
Race	0.037	0.916	−0.666 to 0.741	0.337	0.475	−0.597 to 1.272
Sex	−0.234	0.448	−0.844 to 0.376	0.111	0.791	−0.718 to 0.939
Baseline mRS	0.629	**0.002**	0.236–1.022	0.575	**0.036**	0.039–1.111
Collateral grade	−0.668	**0.014**	−1.194 to −0.141	−0.785	**0.033**	−1.506 to −0.063
Discovery to recanalization	0.0003	0.555	−0.001 to 0.001	0.001	0.339	−0.001 to 0.002
Outside hospital	−0.356	0.294	−1.026 to 0.314	0.304	0.517	−0.624 to 1.231

*Bold values indicates statistical significance p < 0.05*.

## Discussion

Currently, IV tPA prior to mechanical thrombectomy (MT) is considered standard of care for eligible patients presenting with LVO within the accepted time window (4.5 h from last known normal) (10, 11). Nonetheless, the added benefit of combination therapy has been questioned and concerns raised regarding potential drawbacks. Several trials have recently attempted to address the role of tPA in MT with mixed results. DIRECT MT was a randomized controlled clinical trial of 41 Chinese centers that found no difference in 90 day mRS score for patients bridged with IV tPA vs. treatment with MT alone; (19) however all patients included presented directly to the thrombectomy-capable center, and a similar trial was unable to show non-inferiority of MT compared to the combined approach. (20) While we did not find major significant differences in radiographic or functional outcomes between patients presenting to the ED vs. an OSH, those transferred did require fewer passes to achieve recanalization, which may have been in part due to the fact that there was a longer period of time for tPA to soften the clot. Previous studies have shown the rate of recanalization after IV tPA, negating the need for subsequent intervention, to be as high as 11% (21); however, a recent meta-analysis showed a lower rate of recanalization following IV tPA for ICA terminus and proximal MCA occlusions (22). More recently, Ren and colleagues reported that treatment with IV tPA prior to MT led to distal clot migration and a higher rate of unsuccessful clot removal in their patient population (9).

Given that the administration of IV tPA takes time, appears to have varying degrees of effectiveness, and theoretically increases the risk of clot migration and hemorrhagic transformation, some have raised the concern that using IV tPA as a bridge to MT may actually prolong recanalization times and be harmful to patients (23). Our data support that IV tPA does lead to a higher rate of distal clot migration; however, this overall rate is relatively small, and did not reach statistical significance in our population, consistent with at least one prior study failing to demonstrate a significant association between IV tPA and thrombus migration (23). Interestingly, at 13%, our rate of partial recanalization following tPA was lower than those reported by Seners and colleagues (up to 33%) (24), but more in line with the 7% rate reported by Mendez et al. (25). The variability may have in part been influenced by thrombus location, as the majority of our occlusions involved the distal ICA and proximal MCA. More importantly, despite a higher rate of clot migration with tPA, we show that taking the time to administer alteplase neither significantly increased door to groin puncture times nor interfered with clot removal, and actually improved reperfusion (TICI scores), perhaps by softening the thrombus and making it more amenable to intervention. Time from groin puncture to recanalization was not affected by treatment with tPA, however the number of required passes to recanalize the vessel was lower, particularly in OSH transfers, perhaps because there was a longer period of time for tPA to take effect (26, 27).

Notably, the rate of symptomatic hemorrhage was not increased, even when times were longer, to potentially offset this advantage. One of the most feared complications, studies have shown that longer times to reperfusion are associated with higher bleeding rates (28), so this is an important finding when considering the risk/benefit profile of treatment. Our overall, rate of sICH (14%) is similar to that of other studies for mechanical thrombectomy (29) and is as expected higher than that for administration of IV tPA alone (1). Patients within our cohort were more likely to experience sICH if they were white and had larger infarct volumes, though other variables such as age, atrial fibrillation (which can lead to larger infarcts), hypertension, and longer times to recanalization trended toward higher hemorrhage rates and may have reached significance with a larger sample size. This is also consistent with the literature (29, 30) and did not vary based on whether they were bridged with IV tPA prior to thrombectomy. We did observe a slightly higher sICH rate in patients from an OSH who were not bridged with IV tPA. This may have been due to higher rates of systemic anticoagulation in this group. Interestingly, the use of stentrievers vs. mechanical aspiration led to more hemorrhages in our cohort. This difference did not reach statistical significance, but may be at least in part due to success of reperfusion and reinstating blood flow (more TICI 3 vs. 2b seen with stentrievers), as better TICI scores was also associated with higher hemorrhage risk, which theoretically could be due to increased risk of short-term reperfusion injury in those recanalized vs. those whose vessel remained closed. Importantly, adequate reperfusion (2b/3) was required in order to achieve a good outcome at 90 days.

In addition to clot migration and hemorrhage risk, we evaluated the effect of combined therapy vs. MT alone on long-term functional outcome (mRS). Similar to previous studies (31, 32) our data suggest a significant recovery benefit when tPA is given prior to MT. The difference persisted even when adjusting for time to recanalization and other differences between the two groups. While the underlying mechanism remains unclear, it has been consistently demonstrated. One possibility is that early administration of IV tPA leads to clot migration or partial recanalization that could contribute to earlier or increased perfusion to salvageable brain during the intervention period, allowing for a better long-term prognosis. More work is needed to elucidate the underlying mechanisms.

Our study is not without limitations. It is a relatively small cohort from a single institution and is not randomized, introducing the possibility that those not treated with IV tPA had worse outcomes because of additional comorbidities or circumstances that prevented them from being tPA candidates, including delayed presentation from symptom onset. To account for this, we adjusted for the most common exclusion criteria, time to reperfusion, and compared functional outcomes for only those treated within the early window (<6 h); however, there may be additional confounding factors. In addition, the average door to puncture time was >2 h. It is possible that centers with shorter times would find that IV tPA administration does prolong time to groin puncture. Door to scan, door to needle, and door to puncture times were only calculated and analyzed for patients presenting to our ED, rather than those transferred given the information available to us, so time from symptom onset to reperfusion was used evaluate the impact of time on risk of clot migration and sICH.

Despite these limitations, our data are consistent with other subgroup analyses indicating that administration of IV tPA improves MT outcomes, and we show that this does not come at the expense of prolonged treatment times, procedural difficulties, or higher hemorrhage rates. Notably, despite some differences, we did not find enough variance between individuals presenting directly to a thrombectomy-capable center vs. being transferred for the procedure to advocate for different treatment paradigms based on site location, and all patients appeared to benefit functionally from bridging with IV tPA prior to thrombectomy.

## Conclusion

For patients undergoing MT for large vessel occlusion, the use of IV tPA to bridge to MT does not delay treatment times or result in increased clot migration leading to difficulty with clot extraction or higher rates of intracranial hemorrhage. Treatment with IV tPA at both thrombectomy-capable centers and transferring hospitals results in better overall TICI scores and long-term functional outcomes than those treated with MT alone. When possible, use of IV tPA in combination with MT should remain first line treatment for large vessel occlusions.

## Data Availability Statement

The raw data supporting the conclusions of this article will be made available by the authors upon request, without undue reservation.

## Ethics Statement

The studies involving human participants were reviewed and approved by The Johns Hopkins Institutional Review Board. Written informed consent for participation was not required for this study in accordance with the national legislation and the institutional requirements.

## Author Contributions

AC and EB were responsible for data collection and drafting the initial manuscript. EL was responsible for data collection. OI was responsible for overall conceptualization of the project, data collection, and manuscript revision. EM was responsible for overall conceptualization of the project, oversight, data analysis, and manuscript revision. All authors contributed to the article and approved the submitted version.

## Conflict of Interest

The authors declare that the research was conducted in the absence of any commercial or financial relationships that could be construed as a potential conflict of interest.
